# Improvement science as the engine for the next generation of learning health networks

**DOI:** 10.3389/frhs.2026.1806983

**Published:** 2026-05-06

**Authors:** Gareth J. Parry, Lloyd Provost, Peter Margolis

**Affiliations:** 1 Health Evaluation Research Lab, Cambridge Health Alliance, Cambridge, MA, United States; 2 Department of Psychiatry, Harvard Medical School, Boston, MA, United States; 3 Associates in Process Improvement, Austin, TX, United States; 4 Stanford University School of Medicine, Palo Alto, CA, United States

**Keywords:** causal inference, implementation science (MeSH), improvement science, learning health network, learning health systems, quality improvement

## Abstract

While the evidence-based care movement has traditionally relied on randomized controlled trials (RCTs), these methods often lack the flexibility and external validity required for complex, real-world clinical settings. To overcome these issues, this paper proposes an Improvement Science Informed Learning System (ISILS) for Learning Health Networks. The ISILS operates as a continuous, self-reinforcing cycle. Its design integrates methods for identifying and overcoming unwanted variation in outcomes, determining effective practices, and rapidly implementing those successful practices across network member systems. This actor-oriented organizational structure promotes knowledge creation across diverse sites, enabling patients, clinicians, and researchers to co-produce health. Using the management of pediatric Crohn's disease as an illustrative case, this paper illustrates how the ISILS can identify superior therapeutic drug monitoring strategies and improve network-wide remission rates. Ultimately, this integrated approach ensures that best-practice care is a predictable output of a designed and active learning system.

## Introduction

Future high-reliability health systems depend on transitioning clinical practice into a system where care delivery evolves through continuous, evidence-based improvement. We describe recent efforts to achieve this vision, including the application of clinical research, improvement science, implementation science and Learning Health Systems and Networks. We propose an Improvement Science informed Learning System (ISILS) that ensures best-practice care is a predictable output of active Learning Health Networks.

### Learning health systems

Emerging in the 1990s, the evidence-based care movement aimed to shift clinical practice toward decisions supported by the best scientific evidence. This movement was dominated by the findings derived from randomized controlled trials (RCTs). Although RCTs are the gold standard for establishing efficacy, they often operate under highly controlled conditions, not always reflective of the complexity of real-world clinical settings. This often led to situations where highly protocolized interventions, inflexible to local adaptations, failed to achieve expected benefits when moved into routine care. Also, since the 1990s improvement science, applied through practical quality improvement (QI) methods has been widely used to enhance clinical practice, and make the reliable provision of evidence-based care more tailored to local systems. More recently implementation science has grown, aiming to provide generalizable strategies and approaches for implementing evidence into practice.

To address the gap between evidence generation and its systematic application, the Institute of Medicine introduced the Learning Health System (LHS) concept in 2007 (1). The LHS integrates research and practice, with knowledge generation a continuous cycle where clinical data collected during routine care informs new research questions, the findings of which are rapidly tested and then implemented back into practice, ultimately improving patient care. This model creates a learning feedback loop, explicitly acknowledging the need for improvement science, using rapid-cycle testing. Additionally, implementation focuses on the systematic uptake of research findings and evidence-based practices into routine care. The LHS serves as an overarching structure for integrating the separate yet complementary scientific areas of clinical research and the practice-oriented fields of improvement and implementation science. Indeed, Johns Hopkins' Berman Institute of Bioethics “Common Purpose” framework for Learning Health Systems shifts the ethical focus from the research/practice divide to a core moral commitment to continuous learning from care delivered, with the purpose of improving future patient care (2).

A recent systematic review, despite acknowledging various advances, concluded that most existing LHS remain more aspirational than truly realized (3). Moreover, single institutions often lack the number and diversity of cases to solve complex, rare, or population-level problems (4, 5). This limitation may be overcome by consolidating learning across multiple systems, establishing Learning Health Networks (LHNs) (6).

### Learning health networks

LHSs operate at scales varying from individual relationships between clinicians and patients to large formal health care systems (7). LHNs were designed specifically to improve care across multiple organizations who are not always jointly governed. Thus the infrastructure required to support LHNs differs from that required to support a LHS. The LHN has been defined as a “multisite, practice-based clinical network that uses data for research and improvement” (8). LHNs are particularly important in clinical field with a small number of cases, for example rare diseases (9). The LHN's goal is to create a collaborative learning environment transcending institutional boundaries to address complex care problems.

A core design component of many large-scale improvement initiatives is facilitating those at the point of care to learn directly from each other (10, 11). The shift toward Learning Health Networks (LHNs) represents a move from “passive observation” to “active co-production.” However, for these networks to function, they must actively learn and then spread, adapt and scale what works across sites (12).

By connecting multiple sites, an LHN can collate resources, knowledge, and know-how that no single site possesses. LHNs aim to create a practical and applicable knowledge management system allowing for innovation and the spread of evidence across geographic and organizational boundaries (13). With this collaborative system, a successful intervention developed in one location can be quickly adapted and implemented elsewhere.

Successful LHNs like ImproveCareNow or the National Pediatric Cardiology Quality Improvement Collaborative are developed using an actor-oriented network architecture (14). Networks facilitate flexible interaction among people, places, and things (e.g., patients, clinicians, researchers, organizational entities, and databases). A network is composed of nodes or “actors” and the links that connect them. LHNs are actor-oriented co-production systems, fostering collaborations among network actors, including engaged patients, families, clinicians, staff, scientists, and communities. Developing these network systems adds complexity beyond that of a single-site system. Here, network actors review data, often in the form of Shewhart charts, to identify gaps in patient care and outcomes, and prioritize areas for improvement (15, 16). LHNs function on the knowledge commons principle (17, 18). LHNs use transparent data sharing to drive learning, innovation, and priority setting. This transparency, facilitated by review of unblinded comparative performance data uses social pressure and gamification to motivate improvement (19).

### Improvement science

At the heart of LHNs is the use of improvement science to drive improvement in outcomes. Clinical research has often been characterized as asking questions primarily focused on “Does this drug work?”, and improvement science as focused on asking questions such as “How do we get this drug to the appropriate patients at the right time every time?” (20) Improvement science can impact much more. Perla et al. expanded on this characterization to describe the science of improvement as an interdisciplinary field focused on developing and applying knowledge to improve systems (21). They argue that improvement is not just a set of tools (like Lean) but a rigorous scientific discipline grounded in philosophical and theoretical foundations. They define improvement science as the interaction of systems thinking, understanding variation, the theory of knowledge, and psychology applied to improve the performance of processes and systems. They emphasize that it is an applied science, learning how to apply subject-matter knowledge to achieve improvement in care processes, and patient outcomes in diverse, real-world contexts (22). Improvement science includes all phases of system change: developing, testing, implementing, and scaling and spreading changes. A core foundation of improvement science, conceptualistic pragmatism, says you only need enough data to make a reasonable prediction for the next test, guiding practitioners from analysis paralysis, or waiting for perfect data before acting (20, 23). Additionally, conceptualistic pragmatism requires you to state *why* you think a change will work before you try it, avoiding haphazard changes without a theory. In other words, they describe improvement science as the science of learning by doing, where your best theory informs a prediction, you act on it, and you let the results of that action refine your theory. This approach aligns with the Scientific method that underpins clinical research (24).

Improvement scientists use multiple methods for learning and LHNs offer the opportunity to use sophisticated PDSA's incorporating planned experimentation with multi-factorial to identify systems-focused changes that lead to or cause the most desirable outcomes (25). Such approaches are based on the work of Fisher, where a two-armed randomized controlled trial is the simplest type of factorial design (26). Recently, Neuhauser and colleagues revisited the contributions of Bradford Hill and RA Fisher to clinical research arguing for the use of factorial designs in the form of planned experimentation to identify the optimal combination of approaches for improving patient outcomes (27). This suggests improvement science can align with clinical research to provide methods and approaches to rapidly move from scientific discovery, through to effectiveness studies and implementation (22).

### Implementation science

In 2006, in the inaugural edition of the journal Implementation Science, the field was defined by Eccles and Mittman as “the scientific study of methods and strategies that support the uptake of evidence-based practices into regular use” (28). Implementation science aims to produce generalizable scientific knowledge regarding the universal principles of adoption. Since then, implementation science has solidified as a field, where frameworks such as CFIR, ERIC and tools including implementation research logic models and process mapping have emerged (29–32). Their application has enabled researchers to identify principals and strategies with the potential to align with the context and characteristics of an intervention increasing the chances they can be spread or scaled-up across multiple settings (33). An implementation science taxonomy has emerged, and is routinely used by funding agencies, such as the NIH (34, 35). These include the use of hybrid designs to clarify whether the intervention, the implementation strategy or both are the subject of a study.

Implementation of tested changes into a system was an established component of improvement methods prior to the emergence Implementation Science. The focus on systems change is vital to the successful implementation in an LHN environment. Consequently, in LHNs, Implementation science can add rigor to spreading or scaling-up evidence of what works. Improvement science is still required to test what system-level changes are required to embed a new intervention into a new setting and increase the chances that improved outcomes are sustained (35–37).

### Causal inference in learning health networks

When comparing treatment effectiveness, RCTs assigning patients randomly to therapies are viewed as the gold standard (38). The RCT is a causal inference method, using randomization to isolate the effect of an intervention from other confounding influences. The improvement science approach of planned experimentation, expands on two-armed RCTs, providing methods for factorial designs identifying what combination of changes causes an improvement (25, 39). For example, Piazza and colleagues conducted the Slug Bug study, aiming to reduce central line associated bloodstream infections (CLABSI) in neonatal intensive care units. Four existing CLABSI prevention strategies formed a 4-factor design, with hospitals assigned to one of the resulting (2^^4−1^) 8 groups. The combination of sterile tubing change and hub scrub compliance monitoring were associated with the lowest rates of CLABSI. Follow up implementation across sites validated the effect of sterile tubing change (40). Thus, planned experimentation provides a complementary causal inference approach, using planned variations to identify which system-level changes cause improvement.

LHNs typically generate large longitudinal data, where patients are assigned to treatments and therapies based on current medical practice and systems. Treatment comparisons using such observational data with multiple regression techniques are usually biased. Designs based on interrupted time series, propensity scores and discontinuity regression have been proposed as ways to address this (41, 42). Additionally, when assessing a treatment strategy, the treatment in one time period may depend on the health status of the patient in the prior time period, which in turn may depend on the choice of treatment in the time period before that (see case-study in the section below). This leads to “confounding by indication”, which regression methods cannot account for.

**Figure 1:**
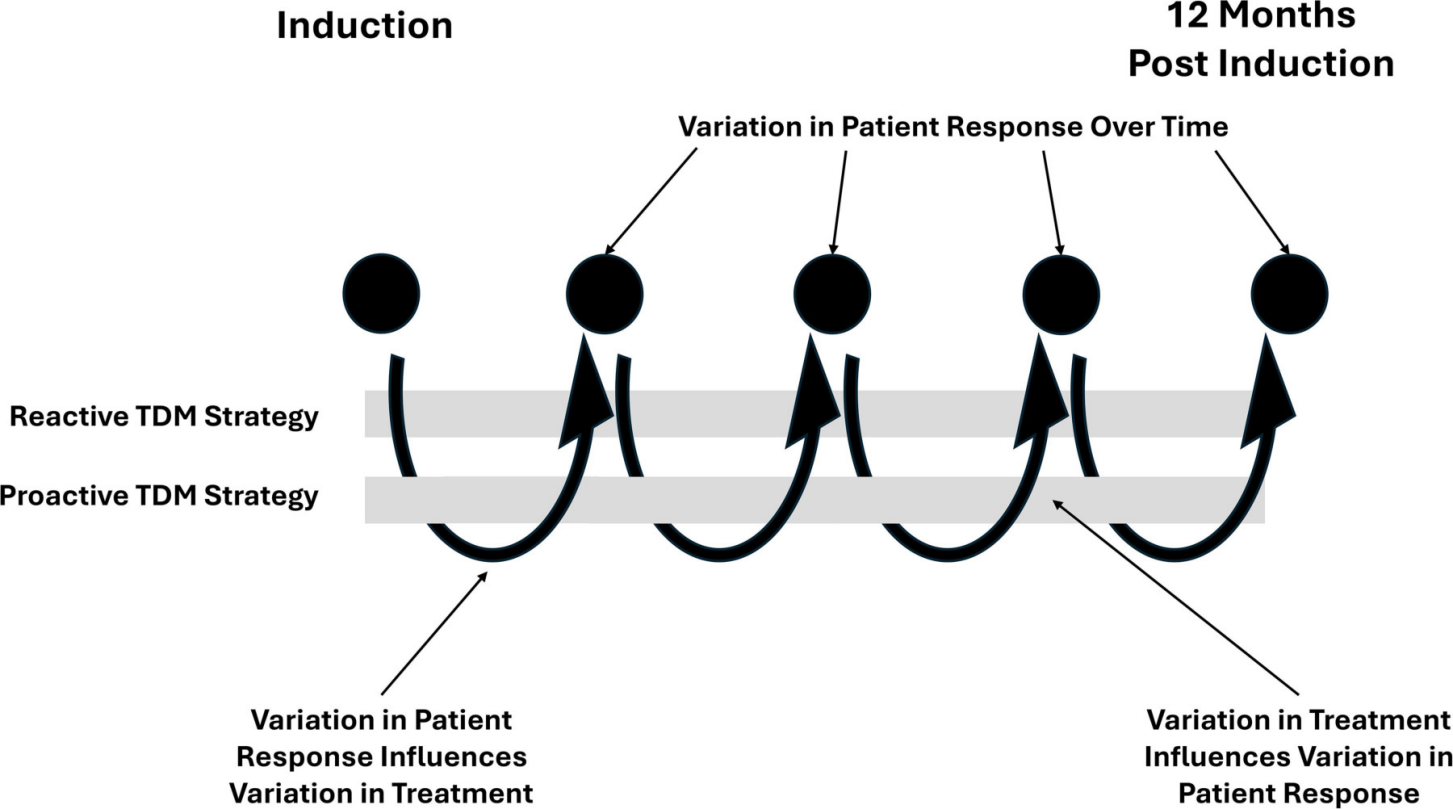
The relationship between practice patterns and improvement in remission rates in the treatment of Crohn's disease over the life of the network.

Recently developed statistical techniques can overcome the limitations of regression methods, allowing observational data to approximate the causal rigor of RCTs (43, 44). Specifically, Longitudinal Targeted Maximum Likelihood Estimation (LTMLE) employs machine learning to balance patient characteristics across comparison groups over multiple timepoints (45). Utilizing non-parametric machine learning, making no assumptions about data distribution, the predicted difference in outcomes between groups is doubly robust (46). LTMLE enables study of dynamic treatment strategies, adapting to a patient's changing clinical status over time. Unlike traditional RCTs focusing on fixed treatments, LTMLE evaluates personalized, rule-based approaches, such as adjusting drug doses in pediatric Crohn's disease based on therapeutic drug monitoring. This method better reflects real-world clinical decision-making, enabling predictions of patient outcomes for adaptive regimens. By combining LTMLE with target trial emulation, the rigor of RCTs can be approximated (43). For example, Figure 1 illustrates for patients with Crohn's disease a comparison between reactive dose adjustments (only when symptoms appear) and proactive adjustments (aiming for specific drug levels regardless of current symptoms), highlighting how individual treatments change with patient response. LTMLE incorporates these variations to predict remission rates after 12 months.

### The improvement science informed learning system (ISILS) for LHNs

A successful LHN requires an active learning system to be embedded. We propose this learning system is informed by Improvement Science and flows seamlessly, integrating methods that identify and reduce variation, identifying what works and rapidly implementing this learning across member systems.

**Figure 2:**
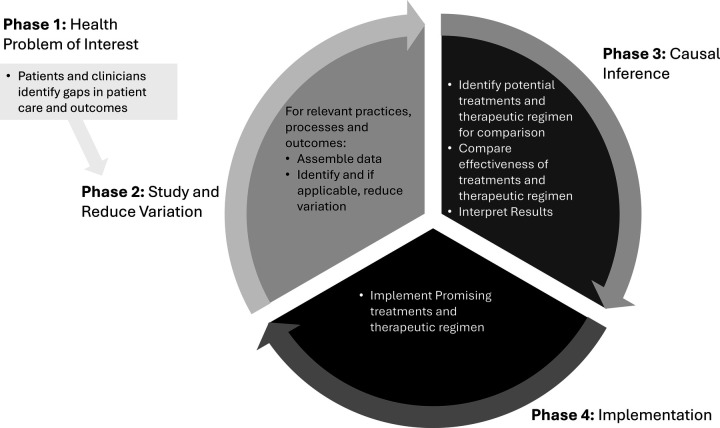
The improvement science informed learning system for learning health networks.

Our proposed Improvement Science Informed Learning System (ISILS) including components of causal inference and implementation science is illustrated in Figure 2. Drawing upon the framework articulated by Friedman, the ISILS provides a conceptual model integrating multiple disciplines (47). The ISILS is a dynamic, continuous, and self-reinforcing spiral, driving continuous improvement and knowledge generation. This cyclical nature ensures that data informs interventions that are evaluated for causal effects, and successful strategies are systematically implemented, closing the loop and feeding new insights back into the system for further refinement and learning. The ISILS comprises:

1. Phase 1: Health Problem of Interest. Patients and clinicians identify gaps in patient care and outcomes.
2. Phase 2: Study and Reduce Variation. Relevant data on practices, processes and outcomes related to the health problem of interest are assembled. Shewhart charts and QI methods are used to learn how to make processes stable and reproducible.
3. Phase 3: Causal Inference. Analysis is planned and undertaken identifying “what works”.
4. Phase 4: Implementation: Implementation strategies are developed to spread and scale up “what works” into the day-to-day processes systems network-wide and to validate learnings from the implementation network-wide.
5. Phase 5: Return to Study and Reduce Variation. The cycle returns to Phase 1), analyzing data to determine whether practice and processes have become more reliable and outcomes have improved.

Table 1 summarizes the ISILS and provides example of where each phase has been successfully applied.

**Table 1:** The improvement science informed learning system for learning health networks – phases and examples.

Phase		Examples
1) Health Problem of Interest.		
	Patients and clinicians identify gaps in patient care and outcomes	• ImproveCareNow (48, 49) • PEDSnet (50) • Fontan Outcomes Network (51)
2) Study and Reduce Variation		
	Relevant data on practices, processes and outcomes related to the health problem of interest are assembled. Shewhart charts and QI methods are used to learn how to make processes stable and reproducible.	• Intermountain Healthcare (52) • Keystone ICU Project: Michigan (53) • Systematic Review of the use of Statistical Process Control in Healthcare (54)
3) Causal Inference		
	Analysis is planned and undertaken identifying “what works”.	• Slug Bug Study (40) • Pediatric Intensive Care Feeding Strategies (55) • Treatment Frequency and Suicidal Behavior (44)
4) Implementation		
	Implementation strategies are developed to spread and scale up “what works” into the day-to-day processes and systems network-wide and to validate learnings from the implementation network-wide.	• Project JOINTS (56) • Keystone ICU Project: Michigan (53) • WHO Safe Childbirth Checklist: Ethiopia (57)
5) Return to Study and Reduce Variation.		
	The cycle returns to Phase 1), analyzing data to determine whether practice and processes have become more reliable and outcomes have improved	• Intermountain Healthcare (52)

### ISILS case study

While Table 1 outlines settings where each phase of ISILS has been applied, a hypothetical example in managing pediatric Crohn's disease can further illustrate its use.

### Phase 1: health problem of interest

Patient outcomes for the treatment of Crohn's disease are reviewed by patients, families, and clinicians to identify gaps in care. They identify that while anti-TNF therapies are effective, many children experience a loss of response over time, leading to disease flares. The network learns that no consensus exists on the best “therapeutic regimen” for applying TDM for adjusting drug doses based on blood levels to maintain remission.

### Phase 2: study and reduce variation

Before testing complex therapeutic strategies, teams ensure that delivery systems are stable and reproducible. The network gathers site and patient-level data on treatment, processes, and outcomes. They use Shewhart charts to identify variation in how often clinicians order TDM and how they respond to low drug levels. This QI process, in collaboration with patients, families and clinicians results in standardization and greater reliability of basic processes.

### Phase 3: causal inference

In the causal inference phase, data on practice variation can identify potential treatment and alternative therapeutic regimens. In our example, the network identified that of patients receiving TDM, over 80% receive care aligning with one of two rule based strategies – Reactive TDM, where the biologic dose is adjusted only when a patient shows symptoms; or Proactive TDM, where the dose is regularly adjusted to maintain a specific target drug concentration, even if the patient feels well. In both strategies, clinicians routinely adjust treatments based on the response of the patient. In collaboration with the patient and family advisory group, the network decides to undertake an analysis to compare 1 year remission rates in patients who receive the Reactive TDM vs. Proactive TDM using an LTMLE approach. The results find that Proactive TDM leads to 20% higher remission rates than Reactive TDM.

### Phase 4: implementation

Insights from the learning derived in Phases 1–3 are used to develop implementation strategies to support sites in adopting and implementing Proactive TDM into their systems. This might involve integrating automated drug-level alerts into the EHR or sharing lessons from positive deviant sites that have already successfully established proactive monitoring within their systems of care. An “all teach, all learn” platform is established enabling sites to learn from each other, building will and motivation through active sharing of challenges, successes, tools and resources (58).

### Phase 5: return to study and reduce variation

The cycle returns to Phase 1, and the network employs Shewhart charts, assessing whether increases in Proactive TDM and a reduction in the network-wide remission rates have resulted.

## Discussion and recommendations

The ISILS described in this paper illustrates how by using the philosophy of Improvement Science, LHNs can become more resilient, self-improving and adaptive systems. The ISILS acts as a blueprint for a high-reliability health system of the 21st century, ensuring that clinical practice evolves into a predictable, designed learning science.

The ISILS utilizes Improvement Science as a feasible operational approach to study variation and stabilize clinical processes across multiple organizations. This stabilization is a methodological prerequisite for causal inference methods to extract clear signals from real-world data.

The introduction of LTMLE to LHN methods provides a novel approach to navigate the complexity of longitudinal clinical care. Unlike traditional regression, LTMLE addresses time-dependent confounding, where a patient's prior treatment influences their future health status, which in turn dictates subsequent treatment decisions. By utilizing Target Trial Emulation, the network can estimate the effectiveness of dynamic treatment regimens with a level of rigor that approximates RCTs.

While causal inference identifies what works, Implementation Science provides a contextual roadmap for how to make interventions work across diverse settings. Within ISILS, implementation strategies are developed to overcome barriers to adoption, ensuring learning from one site is systematically spread across the network.

To apply the ISILS, governance structures and ethical dimensions will need to be considered. The Ethics Framework for the Learning Healthcare System, which outlines seven guiding obligations, offers valuable direction (2). ISILS strongly aligns and encourages the obligation stating that there is an ethical mandate for systems to be designed to learn and to contribute to the purpose of improving the quality of clinical care. In addition, ISILS Phase 1: Health Problem of interest, which identifies areas that matter to patients and clinicians directly aligns with the obligation Respect for dignity (treating patients as partners in learning) and Respect for Clinician Judgment. Some alignment also exists with ISILS Phase 3: Causal Inference on rigorous analysis of observation data and the obligations related to Optimal Clinical Care and Minimizing Non-Clinical Burdens. Other obligations, such as Addressing Inequalities are more subject to external morals and political will.

The ISILS framework also requires capability and capacity to successfully move through the five phases. Although the specifics will vary across LHNs, in general Phase 1: Health Problem of interest will require the establishment of patient-family advisory groups that feel heard and valued. Phase 2: Study and Reduce Variation will require expertise in the use of Shewhart charts and the ability to act on the subsequent learning. Phase 3: Causal Inference requires expertise in Planned experimentation and advanced analytic methods. Phase 4: Implementation requires competence in understanding systems and in developing Implementation strategies. Overall, ISILS will require leadership and governance structures that foster learning and improvement across members of an LHN.

The future of high-reliability health systems depends on the transition of clinical practice to a predictable learning science—a system in which care delivery evolves through continuous, evidence-based improvement rather than chance or tradition. For the ISILS to provide a dynamic, cyclical, and self-reinforcing approach for realizing this vision, we propose the following next steps:

- Develop LHNs with these features:
  - Actor-oriented networks where patients, clinicians, and researchers collaborate as equal partners in identifying gaps in care and in the knowledge generation cycle.
  - Funding and research focus on multiple sites capable of solving complex clinical problems.
  - Training is provided on the use of improvement science methods, including Shewhart charts, to identify, reduce and name variation in practices and outcomes**.**
- Increase the capacity to undertake casual inference: Grow capacity in the health services research community to add planned experimentation and novel techniques such as LTMLE and machine learning into the standard analytical toolkit to leverage longitudinal observational data effectively.
- Integrate implementation science into learning health networks: Ensure implementation science can leverage learning from improvement science and causal inference to efficiently spread and scale treatments found effective in real-world settings. Additionally, increase the focus of implementation science to develop methods for implementing changes into the system and strengthen sustainability.

Progress on the above steps will help ISILS guide providers on a pathway to ensuring delivery of best-practice care is a predictable output of a purposefully designed learning health network.

## Data Availability

The original contributions presented in the study are included in the article/Supplementary Material, further inquiries can be directed to the corresponding author.
